# Preventing Work-Related Musculoskeletal Disorders in Manufacturing by Digital Human Modeling

**DOI:** 10.3390/ijerph17228676

**Published:** 2020-11-22

**Authors:** Jerzy Grobelny, Rafał Michalski

**Affiliations:** Faculty of Computer Science and Management, Wrocław University of Science and Technology, 50-370 Wrocław, Poland; jerzy.grobelny@pwr.edu.pl

**Keywords:** occupational safety and health, musculoskeletal disorders, digital human models, anthropometry, design

## Abstract

This research concerns the workplace design methodology, involving digital human models, that prevents work-related musculoskeletal disorders (WMSDs). We propose an approach that, in conjunction with one of the classic WMSD risk assessment methods, allows one to simplify simulations in a three-dimensional digital environment. Two real-life workstations from a manufacturing industry were modelled in a 3D Studio Max environment by means of an Anthropos ErgoMax system. A number of simulations show that, for the examined cases, classic boundary mannequins’ approaches can be replaced by using 50th percentile of a population individual, with a minimal impact on the WMSD risk. Although, the finding might not be suitable in all situations, it should be considered, especially where compromise solutions are being sought due to other criteria.

## 1. Introduction

Work-related musculoskeletal disorders (WMSDs) refer to diminishing the functionality or damaging of such human body structures as muscles, joints, tendons, ligaments, nerves, cartilage, bones as well as the blood circulation system. These impairments result mainly from such performed work that requires repetitive manual activities, transporting heavy loads manually, excessive energy expenditure, prolonged static forced body posture, etc., and immediate work environment conditions [1] (p. 2), [2] (p. 12). It is well known that inappropriate body posture is one of the causes of musculoskeletal disorders. A particularly awkward body posture taken while carrying heavy objects can cause a serious problem. Similarly, minor postural inconveniences repeated hundreds or thousands of times may also deteriorate human health [1] (p. 4). Such ailments can turn into medical pathological problems and physical changes in the human locomotor system.

Usually WMSDs cover both specific medical diseases such as the tendonitis, tenosynovitis, carpal tunnel syndrome and pain felt in various anatomical structures which is not clearly represented in clinical terms, e.g., neck muscular tensions or non-specific lower back pain [2] (pp. 28–29). These types of ailments and medical conditions are the most common health problem in the European Union. As shown in the report of the European Agency for Safety and Health at Work [2], (pp. 13–14), among work-related health problems, musculoskeletal disorders are reported by about 60 percent of all workers in EU countries (data from 2010 and 2015). The highest rates of complaints were recorded in such occupational categories as agriculture, forestry and fishing (69%), machine operators in industry and assemblers (66%) and craftsmen (65%). The most numerous groups among those listed are operators and assemblers. For example, in Germany, the EU’s largest economy, manufacturing companies generate 45% of national income. Major industrial companies of a production nature (e.g., electrotechnical, mechanical, automotive) are characterized by the presence of large areas of assembly workstations. The constant trend towards product complexity and diversity as well as shortening of the product life cycle and reducing of product batches favors the use of manual assembly [3].The health consequences of professional activity in this group, along with production machine operators, across the EU [2] (p. 15) include mainly back pain (55%), pain in the arms, shoulders neck and upper limbs muscles (47%), as well as lower limbs muscles (33%).

For many years, the efforts of researchers analyzing the WMSD area have been focused on ways to minimize them. General knowledge of mechanisms and factors generating ailments [1] (pp. 13–33) allowed, among other things, for the development of a number of methods for risk occurrence and identification. Thanks to this, it was possible to design and arrange workspaces in such a way that minimizes the WMSDs risk. Methods supporting the prevention of WMSDs have been in use for many years. Among the most popular are the Ovako Working Posture Analyzing System (OWAS) [4,5], Rapid Upper Limbs Assessment (RULA) [6,7], and Rapid Entire Body Assessment (REBA) [8,9].

The OWAS method is intended mainly for the risk assessment of WMSDs for physical work. It analyzes the body posture defined by relative positions of its segments and force loads and assigns them to various risk categories. The RULA tool is focused on the analysis of the upper limbs in sedentary posture. It is similar to OWAS, but it more precisely distinguishes between the positions of the hands while performing tasks. The REBA approach combines the perspectives of RULA and OWAS and identifies the risk of WMSDs where the static work load is predominant [8].

The general idea of the WMSD risk appraisal consists of assessing the deviations of individual body segment angles from their natural, neutral values. This evaluation approach is justified, among others, by precise physiological studies and research on perceived discomfort and fatigue. Aaras et al. [10] document significant relations between the magnitude of the hand segments deviations from their neutral positions and the physical intensity of the load on muscles and tendons. Investigations regarding subjective perception of such body postures were undertaken by a number of researchers. For instance, Corlett and Bishop [11] examined welders discomfort and pain located in specific body segments. Drury and Coury [12], in turn, developed a methodology for evaluating overall comfort while sitting in a chair. Bhatnager et al. [13] associated poorer work performance with bigger perceived discomfort, whereas Kee [14] took advantage of the subjects’ perceived discomfort to automatically generate a three-dimensional isocomfort workspace.

Traditional procedures of using the cited methods of assessing body postures most often involve observation and documenting of body segments positions by taking photos or making videos of typical activities. On the basis of such data, the angles between the body segments analyzed in a specific approach are determined more or less precisely. Then, values of the workload and/or WMSDs risk indicators are determined in accordance with the developed procedures. Usually, these approaches also suggest the appropriate type of intervention when unfavorable results are obtained. As a result, corrective actions may include redesigning tools, rearranging spatial relations, or changing work processes organization. Case studies for such analyses and interventions are described, for example, in [15], where authors examined postural behavior while performing repetitive tasks by using photographs and activity sampling techniques. Priya et al. [16] examined body postures also by taking pictures and using Corlett and Bishop methodology [11] while Gómez-Galán et al. [17] used pictures for analyzing postural load during melon cultivation.

There are important limitations of this type of methods. It is rather difficult to precisely determine angles that characterize the body posture. Furthermore, the investigator might have practical problems in achieving anthropometric representativeness of the surveyed persons (probants). Most often, simply an employee currently working in the studied environment is being examined. In light of the increasing incorporation of females into the industrial workforce, anthropometric analyses involving diverse populations are especially important. They may include not only typical data but also specific anthropomorphical proportions and body shapes. Undoubtedly, the practical inconveniences can be overcome, and special modelling needs met by using modern computer systems supporting 3D design together with the existing digital human models (DHMs).

The main research goal is to examine the possibility of simplifying, in a specific context, the workplace design methodology involving digital human models. We propose an approach that allows one to replace classic boundary mannequins with the use of the 50th percentile individual, with a minimal impact on the WMSD risk level. Although the finding might not be suitable in all situations, it should be considered, especially where compromise solutions are being sought due to other criteria.

## 2. Digital Human Models

The concept of DHMs and its first implementations were created in the late 1960s. The basic idea of taking into account human body properties and limitations as fully as possible while designing workstations in CAD systems was, and still is, quite obvious. Including these features in the virtual space before the physical project is created, allows for conducting tests and making appropriate adjustments very fast and at comparatively low cost. The possible changes are limited only by the creativity of the designer.

Of course, a necessary condition for success of such an approach is the appropriate construction of human body virtual models. The digital, 3D mannequins should statistically correctly represent real populations in terms of both anthropometric features, and biomechanical as well as physiological capabilities. The effectiveness and efficiency of such virtual analyses can be increased by automatic generation of various ergonomic assessments incorporated in software that supports DHMs. They may include, for instance, mechanical workload calculations, approximations of the level of postural discomfort or the thermal comfort degree.

The development of the concept and implementation of DHMs has historically been twofold [18]. In the years of 1960–1990, the parallel trends included computer systems that were primarily designed to support static anthropometric analyses and those meant mainly for dynamic processes studies. Within the first area, started already in the early 1960s, SAMMIE [19,20,21,22], Apolin [23,24], and Anthropos [25,26,27,28] systems were created, among others.

Research on dynamic processes’ analyses involving human participation resulted in the development of such programs as CALSPAN 3D CSV [29], ADAMS [30,31], or MADYMO 3D [32,33,34]. Systems of this type were mainly aimed at analyzing crash tests of virtually designed vehicles. Since the 1990s, one can observe the trend of integrating both directions within complex systems and incorporating them into professional CAD software, e.g., Apolinex [35,36,37], Human [38], or 3DSSPP/AutoCAD [39,40]. Reviews of these earlier solutions can be found, e.g., in [41,42]. The most famous modern applications of this type, i.e., JACK [43] (now part of the Tecnomatix software [44]), RAMSIS [45,46], SAFEWORK [47] (now part of the DELMIA 3DExperience software [48]), or Santos [49,50] are constantly being improved. New features, e.g., facilitate analyses with sophisticated methods of dynamic load assessment or support of psychophysiological evaluations by artificial intelligence [50].

In studies of postural loads and WMSD risks, DHM software packages offer a wide range of multidimensional analyzes of processes and workstations. Some of the modern implementations include modules that automatically calculate classic assessments of postural loads (RULA, REBA) or postural discomfort indicators based on empirical formulas. For conducting research and analyses of this type, older systems that are not developed further, are still in use (e.g., SAMMIE or Anthropos). Most of them, apart from representing the anthropometric features of many populations, have built-in WMSD risk analysis tools and, moreover, they are integrated or cooperate with popular CAD systems. A considerable advantage of these programs is also a relatively simple user interface. The simplicity results not only from many years of experience in their applications, but also from a much smaller range of various functionalities compared to solutions aimed at complex dynamic analyses such as JACK, RAMSIS, or DELMIA.

## 3. Applications of DHM

The use of any DHM system for the ergonomic analysis of tools, workstations, or human work processes in view of potential threats such as discomfort, inconvenience, usually consists of:

1. Creating or recreating a work environment in a virtual space.
2. Insertion of a human body model (dummy), which is appropriate in terms of anthropometrical features.
3. Simulating body posture during the most frequently performed work tasks.
4. Generating workload or discomfort assessments, observing potential inconveniences, e.g., related to the field of view, ranges, etc., and performing the risk assessment of WMSDs.
5. Correction of the workstation and its environment aimed at reducing the potential identified threats and removing inconveniences.

Any population representation can be used in this general procedure. In special cases, digital mannequins representing anthropometric characteristics of specific people intended to work in a given environment may be used. Most often, however, ergonomic design consists in ensuring that geometrical features are matched to the potential population of workers in the range from the 5th to 95th percentile of their body dimensions. For example, Deros et al. [51] applied it to assembly line workstation design, Gragg et al. [52] for the virtual vehicle cab, and Michalski and Grobelny for designing emergency-medical-service helicopter interiors [53].

In similar situations, the designer should predict the appropriate ranges of regulation of work-related environmental components or, when necessary, look for compromise solutions for the studied population. In the first case, the standard approach is to use mannequins representing the 5th and 95th percentiles of body dimensions. Most often the body height is applied, but in specific situations. as in the analysis of arm ranges, also individual body segments can be taken advantage of. The compromise solution usually involves an average individual, that is, a human model with anthropometric parameters reflecting the 50th percentile of the given population [54,55,56].

Various types of digital human models were applied for the ergonomic assessment and design of workplaces in different areas. A relatively substantial number of studies were performed within the automotive manufacturing, for instance, examining automotive assembly tasks [40], driver’s seat adjustment ranges [52], driver’s workplace design [57], reach envelopes in the vehicle workspace [58], or lately statistical approaches for predicting postures [59].

Software enabling preproduction analyses of this kind was also used, e.g., in the aviation industry, for emergency-medical-service helicopters [53], digital human modeling applications in aviation [60,61], in a medical field, e.g., in a surgical ward [62], or while designing for the disabled or elderly people, e.g., [63]. For the review, refer to [64].

Among applications concerned with manufacturing, a wide variety of positions were investigated. For example, Grobelny et al. [65] examined painters, fitters, polishers, pressers, technicians, forklift truck operators, and stockroom deliverers; Schall et al. [66] focused on manual material handling by means of transfer carts and performing tasks such as window and door construction; Peruzzini et al. [67] examined pipe external and internal grinding, cleaning, ovalization control, whereas Zhang et al. [68] investigated welders. Studies directly involving assembly works were conducted, among others, by [69,70,71]. The present study may be treated as a continuation of the trend related to these investigations. A comprehensive review of applications and trends of DHM systems in the manufacturing industry was provided by Zhu et al. [72]. The hardware and software related to area was, in turn, were reviewed by Mgbemena et al. [73].

## 4. Case Studies

### 4.1. Material and Method

The study presented in this paper covers two real-life manual assembly workstations, existing in a Polish branch of an international company. The company produce, among other things, internal and external mirrors for various types of cars. The enterprise operates for dozens of years and is present in 16 countries worldwide. The Polish branch employs more than 500 workers.

#### 4.1.1. Workstations Characteristics

Both workplaces are operated alternately by men and women. The construction of the stands does not allow for the adjustment of the position of its components. Therefore, the current research is focused on determining the most important parameters related to the location of individual work environment movable items that will result in the lowest risk of WMSDs for the entire population of potential employees.

The following assembly workstations were investigated:

(a) The station for manual positioning and fixing of elements inside the mirror body.
(b) The station for fixing mirror’s components with a pneumatic screwdriver.

**Figure 1:**
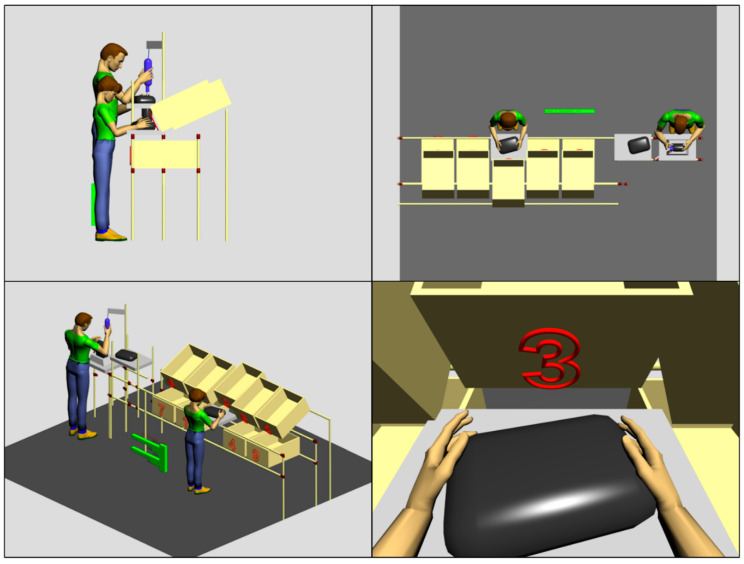
Examined workstations (a) and (b) and basic work posture configurations for 5th percentile of woman and 95th percentile of man while performing basic tasks. The right bottom window presents the field of view of the human model at workstation (a).

Tasks performed on station (a) include manual operations of connecting structural and electrotechnical elements of the mirror with the plastic body. The mirror body is placed on a special stand fixed on the work surface and the individual assembly items are placed in containers behind the work surface. In station (b), the employee places the module completed in station A inside a special holder and tightens, in succession, several screws securing the mirror body parts. Figure 1 shows a 3D model of both stations in a digital 3D space, where all dimensional relationships of the work station environment are kept.

#### 4.1.2. Applied Methodology

The performed analyses were carried out in the Anthropos ErgoMAX system (ver. 6.0.2, HS Group, Kaiserslautern, Germany) which operates within the 3D Studio Max (ver. 6.0, Autodesk, Inc., San Rafael, CA, USA) virtual environment. In the first step, simplified models of the test workplaces and their equipment were prepared in the 3D digital environment. These models precisely mapped essential dimensions of the key workspace components. General, three-dimensional contours were employed to represent work tools and objects.

Figure 1 also illustrates the idea of the simulation research presented in this paper. Digital mannequins representing the appropriate dimensions of the population were generated by the Anthropos software and placed at the virtual stands. Next, the specific body posture taken by employees while performing basic working activities was simulated. Two animation functionalities offered by the Anthropos system were employed for this purpose. The inverse kinematics component and the module for direct setting of angles in joints that connect body segments.

Taking advantage of these precise data and the REBA methodology, the risk level of WMSDs was determined for both examined workplaces. The simulations were performed separately for body dimensions of the 5th and 95th percentiles of the Eastern European population. In the second stage, a procedure for correcting dimensions of the work surface height was proposed. It was aimed at reducing the risk of WMSDs. Detailed research steps are described in the next section.

The workstations modeled in the first stage of the research along with the animated mannequins allowed for the simulation of basic working positions and their evaluation by the REBA method [8,74]. Such an assessment consists of assigning appropriate codes, represented by natural numbers, to the positions of key body segments. Two groups called A and B are distinguished. The A includes the torso, neck, and legs, whereas B comprises arms, forearms, and hands. The trunk movements are divided into four groups depending on its flexion or extension, the neck movements are categorized into two groups in relation to movement angles, also leg positions are assessed in two groups. When it comes to category B, upper arm positions are evaluated according to four different classes, whereas lower arms and wrists movements include two groups. The general principle of coding is to assign higher values to the positions of body segments that deviate more from those favorable from the point of view of biomechanics. More specifically, the scores depend on extension and/or flexions of given body segments. Overall, Group A categories allow for representing as many as 60 posture combinations and class B—36. The determination of the WMSD risk for a given activity comes down to reading the values from table, in which the risk levels are assigned to all 144 combinations of A and B groups’ codes. The result obtained in this way is finally corrected by adding 1 for static work. The static work is defined here as any type of activity in which at least one body segment is held in the same position for at least one minute. All the positions analyzed in this research fulfill this criterion, hence, each overall rating was increased accordingly.

A detailed analysis of the REBA methodology and features of workplaces studied here allowed for a significant simplification of calculations. Employees work on the analyzed workstations in a standing, unforced body posture. This allows for assigning code 1 for the basic torso and leg posture. Admittedly, observation of work tools and objects requires a head tilt, but only in the sagittal plane, without twists or tilting the head to sides. The maximum value of the code for the extreme head tilt is 2. In the cases investigated here, the A value will always amount to 1.

Given the above, our analyses will focus only on employee’s group B segments configurations while simulating work tasks. Since the code for A group equals 1 for our cases, the overall assessment of the risk level is based on the first row of the REBA resulting risk level matrix. The B code, as mentioned earlier, is determined by positions of the arm, forearm, and hand of the more heavily loaded limb.

In the Anthropos system, locations of the main body segments are generated automatically in the form of graphs, showing the percentage deviations of the current position from the neutral position. Table 1 includes the translation of this Anthropos software posture indicator to the angles expressed in degrees and, finally, in the last column, to the appropriate REBA partial codes. The data from Table 1 allow for specifying the general code for part B and are used in further analyses.

In the first step of the analysis, the traditional approach of threshold human models was applied and involved the 5th percentile mannequin representing Eastern European women and the 95th percentile of the male mannequin from the same population. These models were used to assess the risk of WMSDs by the described above REBA methodology.

For both examined workplaces, the body postures for typical tasks were first initially configured by applying the inverse kinematics. This method automatically sets appropriate body segment locations based on the target point indicated by the hand position. This step was followed by precise corrections of the angles in individual joints to obtain final body postures. Flexibility of the employed kinematic chains of human body segments in Anthropos, allows one to simulate limb positions for the same manual task in many ways. Therefore, in this study, we adopted the rule of configuring body segments so that their required final hands locations exhibit minimum partial indicators from Table 1 for the remaining. This means that the presented settings are the least deviating from the optimal ones. Moreover, they are in line with the general paradigm suggesting a relationship between the subjective feeling of postural discomfort and the objective threat of musculoskeletal ailments, e.g., [75].

In our analyses, the position of the line of sight was also simulated and used to ensure that the work tools and items were in the center of the employee’s virtual field of view. A sample of this simulation for a female 5th percentile is shown in Figure 1. Obtained in such a way values of individual body segments angles were the basis of determining partial and overall REBA codes. The figures and tables in the next section illustrate the results of these analyses.

### 4.2. Workstations Analyses, Design Improvements, and Discussion

#### 4.2.1. Workstation (a)—Manual Assembly

**Figure 2:**
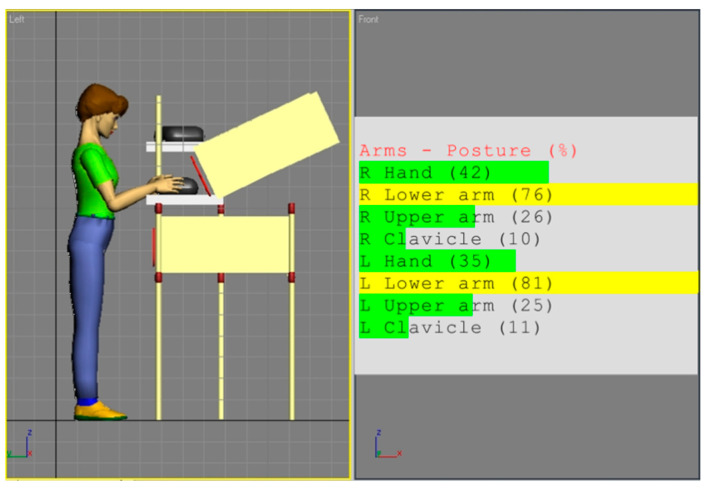
Body posture simulation of the 5th percentile woman during performing basic activities on workstation (a). Angles of the body segment positions expressed as a percentage of maximum ranges are on the image right side.

**Figure 3:**
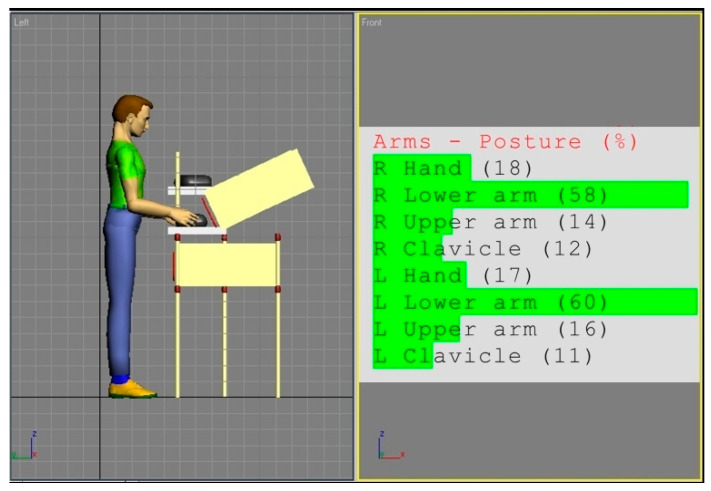
Body posture simulation of the of the 95th percentile man during performing basic activities on workstation (a). Angles of the body segments positions expressed as a percentage of maximum ranges are on the image right side.

Figure 2 and Figure 3 show the simulations of the working postures for a 5th percentile of a woman and a 95th percentile of a man, in workstation (a). The figures also present values of the basic angles for hand segments positions obtained from Anthropos.

The analysis of the partial codes of REBA part B for simulations from Figure 2 and Figure 3 was made in accordance with the data in Table 1 and is put together in Table 2.

According to the REBA methodology, the overall occupational risk assessment for the 5th percentile employee in workstation (a) is 3, and for the 95th percentile one is 2. These codes fall into the second out of five risk categories, where the first one denotes small risk, and the fifth is the highest one. It may be observed that the workplace is designed rather for taller people, however, according to the classification and interpretation of the REBA authors [8], this is not a big risk, but taking corrective actions may be necessary.

**Figure 4:**
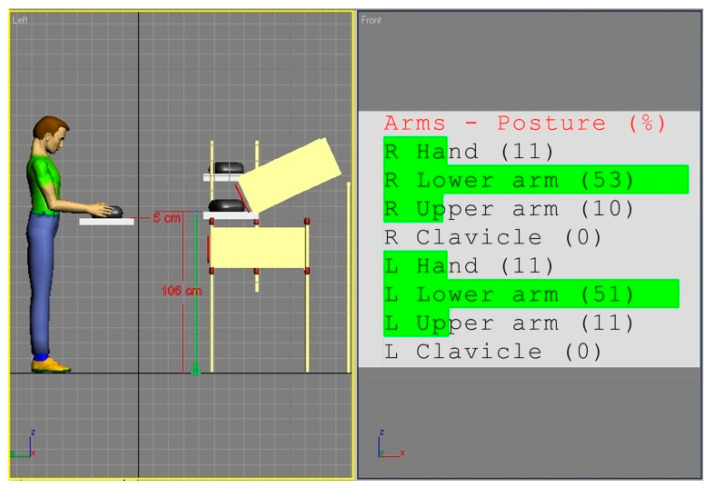
Simulation of modified workstation (a) adjusted to the optimal hand configuration of the 50th percentile mannequin of the Eastern European population. This arrangement’s risk score for Part B of the REBA method is minimal.

In view of the obtained ratings, an attempt was made to correct the workstation (a). The general methodology of the applied improvement approach results from the fact that body segment dimensions in each population are approximately normally distributed. Hence, matching the working environment to average individuals provides, relatively, the largest number of people with good spatial conditions. Since, in the analyzed case, the height of the work surface is the key parameter, a simple procedure was adopted in the simulation studies to correct this parameter. The height of the work surface should be set in such a way that a mannequin, with anthropometric parameters corresponding to the 50th percentile of the adult population from Eastern Europe, could adopt the posture that ensures a minimal risk of WMSDs according to REBA. For this purpose, the dummy was first positioned in such a configuration of the angles of the arm segments that ensured the minimum values of REBA partial codes according to Table 1, and then the location of the work surface was adjusted to this position. The effect of this approach is shown in Figure 4.

The overall REBA rating for the solution from Figure 4 remains at 2, due to static workload but it is the best spatial solution under the existing conditions and constraints.

**Figure 5:**
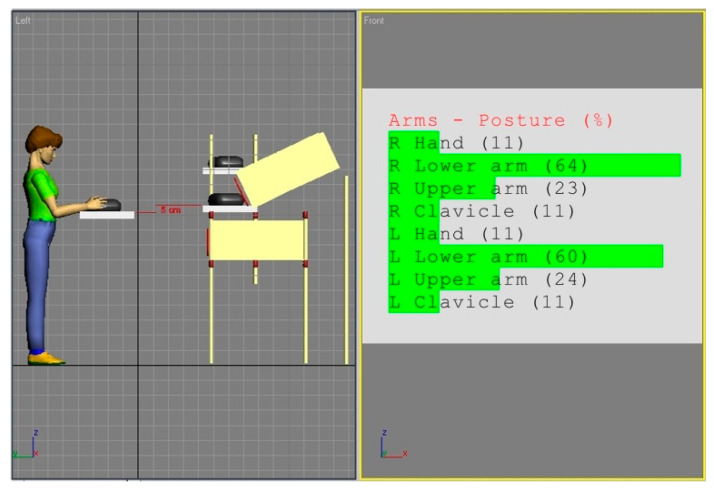
Simulation of 5th percentile woman on the modified workstation (a) adjusted to the optimal hand configuration of the 50th percentile individual.

**Figure 6:**
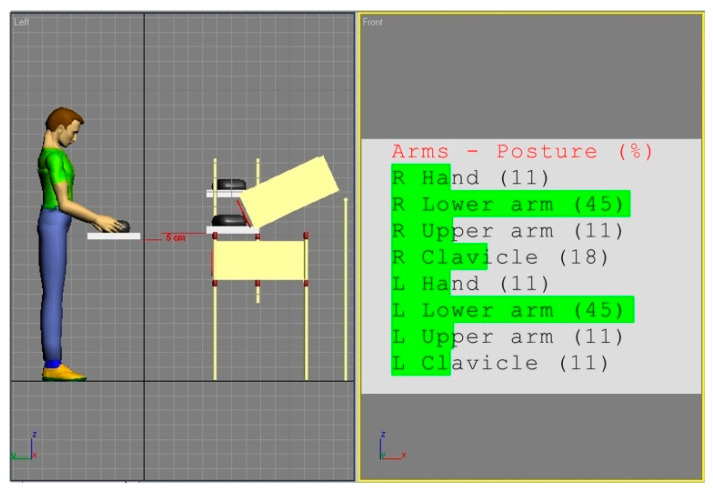
Simulation of 95th percentile man on the modified workstation (a) adjusted to the optimal hand configuration of the 50th percentile individual.

The overall quality of this solution is further validated by simulating the working posture of the extreme digital mannequins and checking the REBA scores once again. The results of this operation are shown in Figure 5 and Figure 6.

Table 3 presents the outcomes of the REBA assessment for the extreme human models placed in the corrected workstation (a).

Calculations from Table 3 show that a slight change of lowering the work surface height only by 5 cm improved the risk category for the female 5th percentile and did not change the risk for the 95th percentile of men. As it is not possible to obtain a lower score for part B of the REBA method, the obtained solution can be considered optimal from the point of view of the WMSD risk, that is, the best under the assumptions made.

#### 4.2.2. Workstation (b) with a Screwdriver

**Figure 7:**
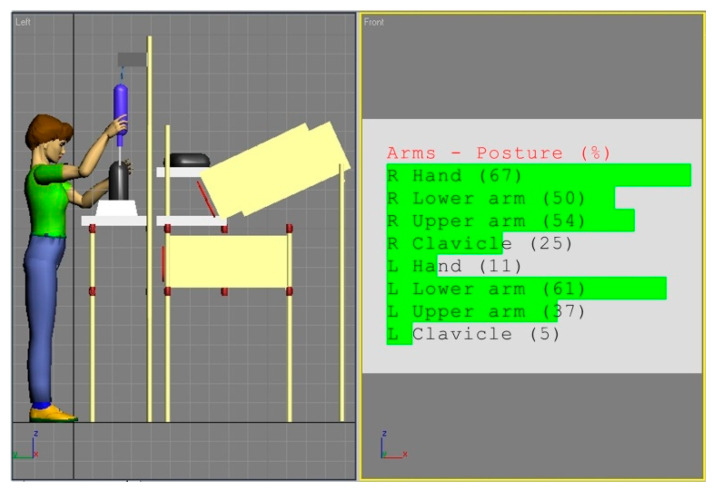
Body posture simulation of the of the 5th percentile woman during performing basic activities on workstation (b). Angles of the body segments positions expressed as a percentage of maximum ranges are on the image right side.

**Figure 8:**
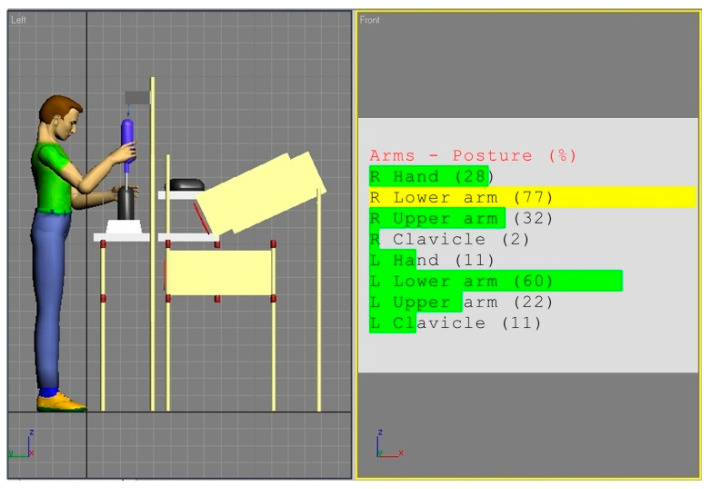
Body posture simulation of the of the 95th percentile man during performing basic activities on workstation (b). Angles of the body segments positions expressed as a percentage of maximum ranges are on the image right side.

A similar procedure was applied for the examination of the workstation equipped with a mechanical screwdriver. Figure 7 shows an existing design analysis for the 5th percentile of a female, whereas Figure 8 presents simulated body posture of the 95th percentile of a male. Both models are from the Eastern Europe population.

The analysis of the angular values shown in Figure 7 and Figure 8 in confrontation with the REBA rules provided the results shown in Table 4.

The results of this analysis indicate that the risk level is average and that appropriate actions are necessary to correct the worker posture. Similar to workstation (a), also here, the work surface is placed too high. Therefore, the risk of WMSDs is especially high for shorter people.

**Figure 9:**
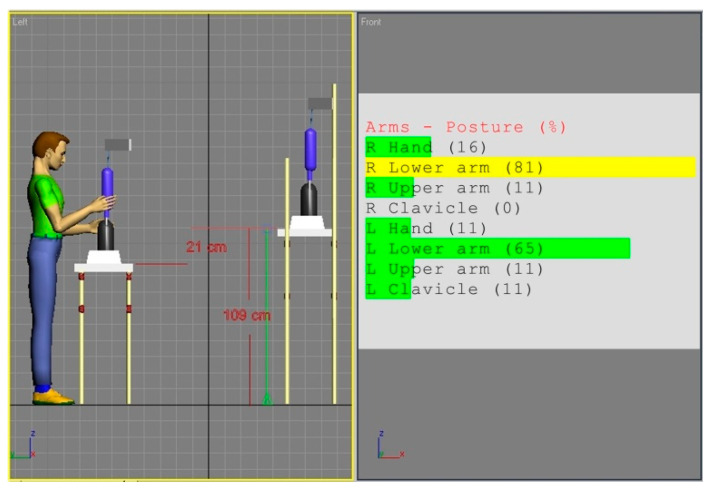
Simulation of modified workstation (b) adjusted to the optimal hand configuration of the 50th percentile mannequin of the Eastern European population. This arrangement’s risk score for Part B of the REBA method is minimal.

As before, adjustments to the workstation spatial arrangement were made based on the optimal settings for the 50th percentile mannequin. In this case, the correction required a significant lowering of the tool holder position. The optimal solution for the average human model is illustrated in Figure 9.

**Figure 10:**
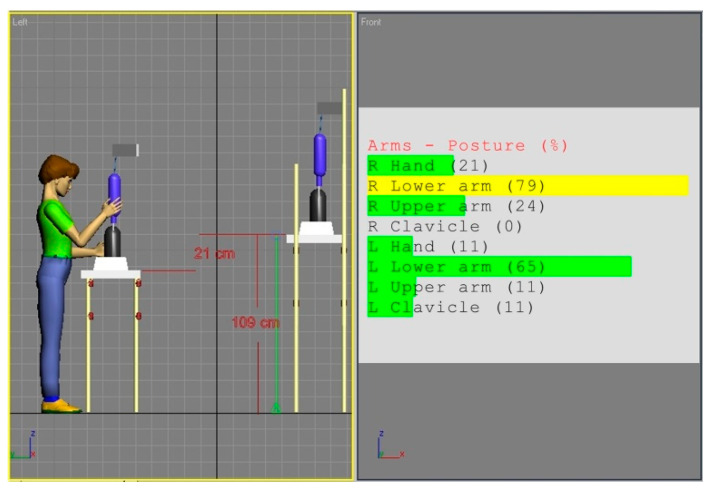
Simulation of 5th percentile woman on the modified workstation (b) adjusted to the optimal hand configuration of the 50th percentile individual.

**Figure 11:**
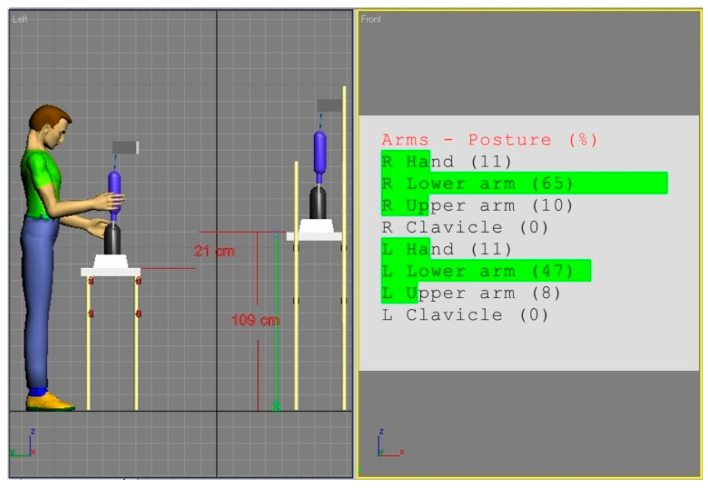
Simulation of 95th percentile man on the modified workstation (b) adjusted to the optimal hand configuration of the 50th percentile individual.

The analysis of the changed design was performed, again, for the threshold representatives of the examined population. The outcomes are shown in Figure 10 and Figure 11.

Table 5 summarizes the REBA risk assessment components for the data obtained in simulations from Figure 10 and Figure 11.

As it can be easily noticed, the application of the strategy of adjusting the height of the work tool to the anthropometry of a 50th percentile individual resulted in a radical improvement in the WMSDs risk level assessments for workstation (b). The solution is almost perfect from the analyzed point of view.

#### 4.2.3. REBA Sensitivity Analysis

**Figure 12:**
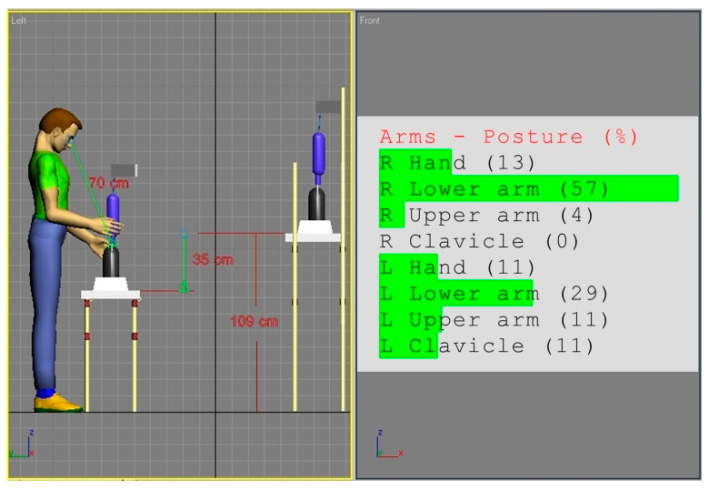
Body posture simulation of the 95th percentile male on workstation (b) designed to be optimal for the 5th percentile female. The distance of 70 cm from employee’s eyes to the work area was measured with the Tape tool.

Even a cursory analysis of the relationships reflected in the matrices of the REBA methodology shows that in manual work, risk assessment is most sensitive to the deviation of an upper arm from its neutral position. Therefore, we examined the solution in which the height of the screwdriver body is optimal in the sense of the upper arm’s position set on the border of its optimal range (i.e., smaller than 11% of the maximal range) for the 5th female percentile. Such an assumption resulted in a radical lowering by as much as 35 cm of the work surface in workstation (b). The akin simulation for the 95th male percentile for this solution is shown in Figure 12.

The outcome of this analysis is surprising because the ideal solution for the 5th female turned out to be also good for the 95th male percentile. The only criterion here is the WMSD risk assessment performed according to the REBA convention. The only doubt diminishing the acceptance of the “for the smallest” design strategy, is the distance between the employee’s eyes and the work items. It is illustrated in Figure 12. In the analyzed workstation (b), such a solution could be accepted because this distance amounts to approximately 70 cm. This is the upper limit of the ergonomic recommendation regarding the placement of visual information that require reading (50–70 cm; Młodkowski, 1998, p. 354; Woo et al., 2016). However, with more precise works, this can be a problem. Especially in assembly works where, apart from manual activities, the visual information processing is also important.

**Figure 13:**
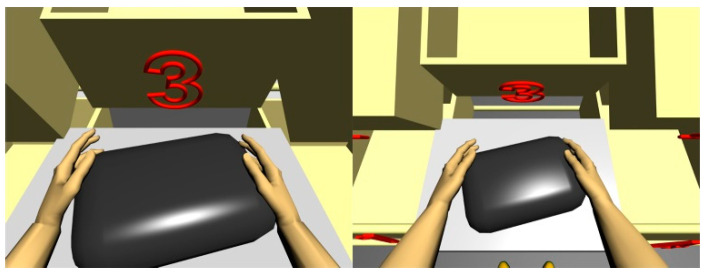
Fields of view of the extreme human models in the basic configuration of the body posture on workstation (a) designed to be optimal for the 5th percentile female. The left image side shows 5th percentile female’s field of view, whereas the right side presents the field of view of 95th percentile male.

In such situations, the arrangement of information components within the employee’s field of view may be crucial for work effectiveness and efficiency. There are no excessive requirements in this respect for the examined workstations. Despite that, fields of view for the extreme mannequins given in Figure 13 illustrate significant differences caused by anthropometric and design differences in this respect.

**Figure 14:**
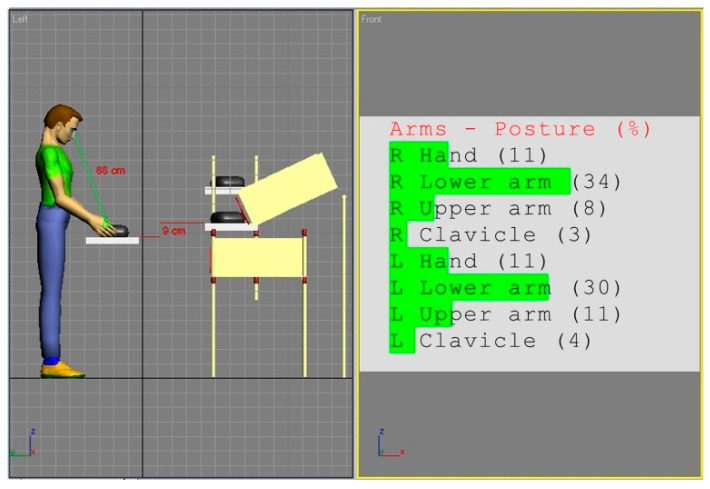
Body posture simulation of the 95th percentile male on workstation (a) designed to be optimal for the 5th percentile female. The distance of 68 cm from employee’s eyes to the work area was measured with the Tape tool.

A similar procedure applied to workstation (a) with an ideal solution for a 5th percentile of a woman, does not change the REBA WMSD risk assessment for the male 95th percentile. In this case, the shift in the worksurface level is small relative to the 50th percentile individual optimization strategy. The simulated posture along with the angle ranges is shown in Figure 14.

In comparison to the simulation results presented in Figure 6, the lower arm angle slightly deteriorated in Figure 14, but the REBA assessment did not change. What is more, the workspace design is now ideal, from the REBA perspective, for the 5th percentile of a woman.

## 5. Conclusions

The presented research results show, above all, the broad possibilities of DHM in the analysis and design of human workplaces. Such analyses seem to be very useful as diversity in the workforce is becoming bigger and bigger due to, among other things, growing proportion of females and people of different ethnic origins. Taking advantage of digital models of workstations and humans is potentially very beneficial both for employers and employees. Though it is, naturally, possible to improve existing solutions by, e.g., providing platforms for shorter people, it is much better and usually cheaper to design the workstations correctly.

Poorly designed workplaces that do not take into account anthropometrical features of different individuals may significantly increase the risk of WMSDs. Improving the workplace conditions is of importance not only from the economical point of view but also from the medical perspective. The higher the risk of WMSDs, the more severe consequences for human health. The list of possible medical problems associated with WMSDs along with their codes from the International Classification of Diseases have been comprehensively listed in [76] (p. 10). The catalog contains as many as 31 disease entities including seven tendinopathies, eight tunnel syndromes and nerve compressions, three hygromas, four bone syndromes, three vascular syndromes, meniscus lesions, and five non-specific disorders.

Although the case study presented here relates to very specific and concrete situations, it seems that the presented results have a significant and potentially universal applications. First of all, they show how many aspects of user-centered design can be addressed using relatively simple DHM software developed, as mentioned earlier, many years ago. The undoubted advantage of the Anthropos ErgoMax system is its implementation in the 3D Studio Max environment. Version 6.0 of this program is easy to learn and use and is completely sufficient for analyzing existing and designing new work environments in terms of their ergonomic properties. The Anthropos software facilitates a flexible insertion of human digital models of many national and regional populations. The inverse kinematics functionality, along with precise positioning of body segments through rotations in joints, allows for performing simulations of any working postures. As it was shown in this study, by combining the features of the 3D Studio Max environment and the Anthropos ErgoMAX system, one is able to obtain detailed data for ergonomic assessments in the areas of anthropometry, fields of view, workloads, or the WMSD risk.

The REBA methodology used here allowed us to significantly improve the designs of existing workplaces in a specific company. Furthermore, the universal finding of the research regards the effectiveness of designing the height of the worksurface for the 5th percentile individual of the population in minimizing the risk of WMSDs for the entire population. As far as we are aware, such a result has not been reported yet in the existing literature. Although the presented approach might not always be suitable, it is worth checking while analyzing workstations in digital environment. Naturally, one also needs to take into account specific limitations, for example, those suggested in this work—the level of work precision or work item visibility in the employee’s field of view.

## Figures and Tables

**Table 1:** Correspondence between the Anthropos posture indicators, expressed as a percentage of the maximum range, degrees of body segments’ flexion or extension, and part B codes of the Rapid Entire Body Assessment (REBA) methodology which was used for assessing work-related musculoskeletal disorders (WMSDs) in the virtual environment. The bigger the REBA code number, the more WMSD risk is associated with the given body segment.

	Anthropos Posture (% of Range)	Position/Movement (Degrees)	REBA Code
Upper arm	<11	Flexion <20	1
	11–25	Flexion 20–45	2
	25–50	Flexion 45–90	3 (+1 if shoulder is raised)
	>50	Flexion 90	4 (+1 if shoulder is raised)
Lower arm	0–50	Flexion 0–60	2
	50–83	Flexion 60–100	1
	>83	Flexion >100	2
Hand	+/−25	Extension/flexion +/− 15	1 (+1 if wrist deviated or twisted)
	>25	Extension/flexion >15	2 (+1 if wrist deviated or twisted)

**Table 2:** Partial and overall codes for REBA part B for workstation (a) analyses involving extreme mannequins.

Body Segment	5th Percentile		95th Percentile	
	Anthropos Posture (% of Range)	REBA Code	Anthropos Posture (% of Range)	REBA Code
Upper arm	26	3	14	2
Lower arm	76	1	58	1
Hand	42	2	18	1
REBA part B		4		1
REBA general (+1 for static work)		3		2

**Table 3:** REBA WMSD risk assessment results for extreme human models on the modified workstation (a) adjusted to the optimal hand configuration of the 50th percentile individual.

Body Segment	5th Percentile		95th Percentile	
	Anthropos Posture (% of Range)	REBA Code	Anthropos Posture (% of Range)	REBA Code
Upper arm	23	2	11	1
Lower arm	64	1	45	2
Hand	11	1	11	1
REBA part B		1		1
REBA general (+1 for static work)		2		2

**Table 4:** Partial and overall codes for REBA part B for workstation (b) analyses involving extreme mannequins.

Body Segment	5th Percentile		95th Percentile	
	Anthropos Posture (% of Range)	REBA Code	Anthropos Posture (% of Range)	REBA Code
Upper arm	54	4 (+1) arm raised	32	3
Lower arm	50	1	77	1
Hand	67	2	28	2
REBA part B		7		4
REBA general (+1 for static work)		4		2

**Table 5:** REBA WMSD risk assessment results for extreme human models on the modified workstation (b) adjusted to the optimal hand configuration of the 50th percentile individual.

Body Segment	5th Percentile		95th Percentile	
	Anthropos Posture (% of Range)	REBA Code	Anthropos Posture (% of Range)	REBA Code
Upper arm	24	2	10	1
Lower arm	79	1	65	1
Hand	21	1	11	1
REBA part B		1		1
REBA GENERAL (+1 for static work)		2		2
